# A Rare Case of Portal Vein Thrombosis Following a Successful Laparoscopic Cholecystectomy

**DOI:** 10.7759/cureus.30294

**Published:** 2022-10-14

**Authors:** Rajan Sood, Mansi Seth, Shivanshu Kundal, Randeep Singh, Bhavnesh Kapoor

**Affiliations:** 1 Department of General Surgery, Maharishi Markandeshwar Medical College and Hospital, Solan, IND

**Keywords:** case report, deranged coagulopathy, laparoscopic cholecystectomy, portal vein thrombosis, portal vein

## Abstract

The function of the portal vein is to drain the blood mainly from the gastrointestinal tract to the liver and its thrombosis is an extremely unexpected outcome of an uncomplicated laparoscopic cholecystectomy. It is believed to be a rarely reported case to date in non-cirrhotic patients. A female patient, aged 43 years, presented to the surgical outpatient department with unexplained severe abdominal pain soon after laparoscopic cholecystectomy. A relative workup was done and radiological evidence revealed the thrombosis in the distal part of the portal vein at its bifurcation which completely occluded the left branch of the vein. Although rare, portal vein thrombosis should be concluded in the differentials for unexplained causes of abdominal pain in the postoperative period of laparoscopic cholecystectomy.

## Introduction

In the new era of laparoscopic cholecystectomy, the necessary learning curve has increased the iatrogenic injuries, most commonly including lesions to the hepatic hilum. Complications from laparoscopic cholecystectomy include injury to the bile duct, intraoperative leakage of bile into the peritoneum, hemorrhage, and injury to the bowel, which could be iatrogenic or due to constraints in minimally invasive techniques [1]. The statistics of the prevalence of portal vein thrombosis in a diseased liver is known to be 0.6-16% and its occurrence post-cholecystectomy is a rarity [2]. Portal vein thrombosis can be an expected outcome in a cirrhotic patient or a patient with a deranged anticoagulant profile. We are reporting a rare case of a female patient, 43 years old, who presented with abdominal pain on the 12th postoperative day of laparoscopic cholecystectomy, diagnosed radiologically as a portal vein thrombosis.

## Case presentation

**Figure 1:**
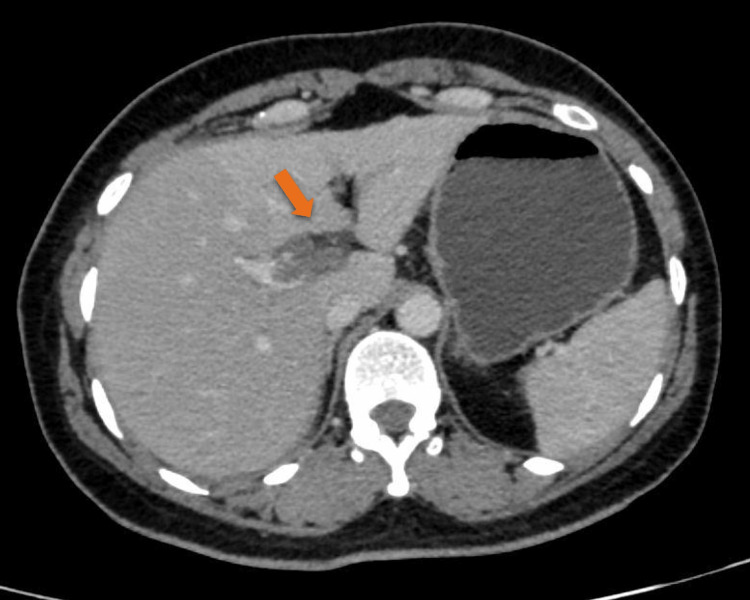
Abdominal CT scan showing thrombosis at the bifurcation of the main portal vein extending to the left branch.

**Figure 2:**
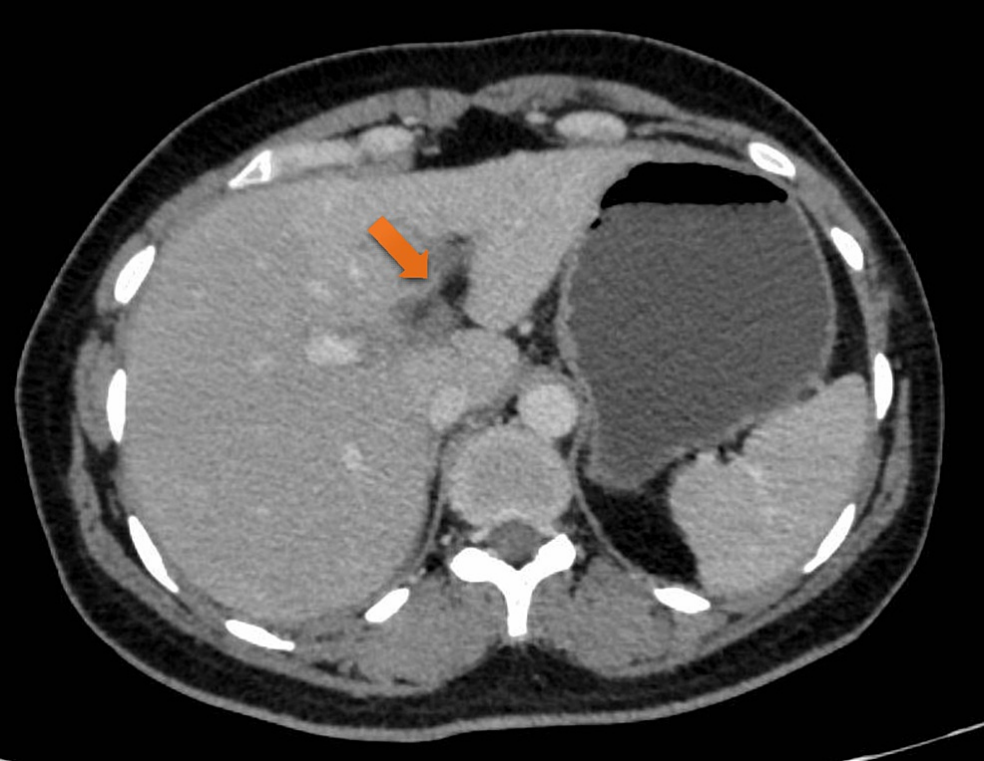
Abdominal CT scan showing a filing defect, indicating blockage of the left branch of the portal vein.

A 43-year-old female resident of Chail, Himachal Pradesh presented to the outpatient department of surgery with a complaint of abdominal pain for two days. The onset of the pain was sudden, continuous, post-prandial, and was present in the epigastric and umbilical region, associated with nausea. The patient had a history of cholelithiasis for which she was operated on 12 days back at our institution and was discharged in a well-preserved and ambulatory condition on the second postoperative day. The patient had no other medical history. There were no intraoperative complications. The immediate postoperative period went uneventful. On examination, the abdomen was rigid and guarded. There was diffuse tenderness present in the abdomen. A provisional diagnosis of acute generalized peritonitis was kept. Chest and abdominal X-rays were normal. Ultrasonography revealed a few dilated small bowel loops and was suggestive of portal vein thrombosis. The findings were confirmed with a contrast-enhanced computed tomography scan that showed thrombosis in the distal portal vein at the bifurcation as shown in Figure 1, and its left branch as shown in Figure 2 as indicated with the arrows. 

Blood test reports showed leukocytosis, increased fasting blood sugar up to 145mg/dl, gamma-glutamyl transferase levels were 90 U/L, alkaline phosphatase levels were 133 IU/L, and decreased albumin to globulin ratio of 1.05. D-dimer value was increased up to 2309 ng/L. Anticoagulant tests namely, Protein C, Protein S, Cardiolipin IgG, IgM, and IgA antibodies, and Factor V Leiden mutation, showed no deviation from the normal results. She was started on 0.6mL enoxaparin injection subcutaneously 12 hourly and was later shifted to tablet dabigatran 150 mg once daily. The patient symptomatically improved within five days of the hospital stay and was discharged with the advice of follow-up after two weeks with a fresh ultrasound of the abdomen. Partial recanalization of the left portal vein was seen after two weeks and the echotexture improved from the previous study. One monthly regular follow-up was taken while the patient continued the tablet dabigatran 150 mg once daily for up to six months. The patient was asymptomatic after the treatment.

## Discussion

The practice of laparoscopic technique is increasing as it provides better cosmetic results and minimal intraoperative hemorrhage. Complications of laparoscopic cholecystectomy include injury to the biliary duct, biliary leakage intra-operatively, hemorrhage, injury to the hollow viscus organs, and rarely portal vein thrombosis [3]. Portal vein thrombosis is a relatively expected complication after surgeries such as removal of colon, appendix, and gastric bypass surgeries. Although rare, only a few cases of the same have been reported till date after uncomplicated laparoscopic cholecystectomy surgeries [4]. The risk of portal vein thrombosis/mesenteric venous thrombosis apart from cirrhosis includes prothrombotic states, pancreatitis, blunt abdominal trauma, use of oral contraceptive pills, obesity, peritonitis, intra-abdominal sepsis, and none of these were elucidated in our patient. Laboratory workup may sometimes show unexpected leukocytosis as in our patient [5]. Most cases point to intra-abdominal infections or malignancy as the underlying cause; however, half of these cases have no identifiable causes. Myeloproliferative diseases such as polycythemia vera and essential thrombocytopenia are known to be associated with portal vein thrombosis [6]. Previously four reported cases of portal vein thrombosis following laparoscopic cholecystectomy in literature have had risk factors such as oral contraceptive pills, diabetes mellitus and coronary artery disease, hypertension, and one patient with no identifiable risk factor. The biochemical markers in these cases were increased IgG, dengue viral fever, low protein S and factor II prothrombin mutation respectively [7]. Workup for the same includes liver function tests, which were deranged, and anticoagulant profile, which was normal. Radiological studies, namely, ultrasonography, magnetic resonance imaging, and computed tomography, visualize the extent of the clot [5]. Heparin followed by oral anticoagulation is used to treat portal vein thrombosis successfully. Anticoagulation aims to prevent further clot extension and favors the recanalization of the portal vessel and it is recommended to be continued for up to six months in uncomplicated cases as in ours [5]. Untreated patients with acute portal vein thrombosis may develop intestinal infarction which leads to intestinal perforation which results further in peritonitis and shock, ultimately causing death [8]. Although rare, portal vein thrombosis should be concluded in the differentials for unexplained causes of abdominal pain in the postoperative period of laparoscopic cholecystectomy.

## Conclusions

Thrombosis of the portal vein is a rare phenomenon to be known in post-cholecystectomy not associated with leading factors such as increased body mass indexed individuals, patients of cirrhosis, and those with no evidence of intra-abdominal malignancy. Although multifactorial, its exact cause is still unknown in patients with a normal anticoagulant profile in pre-operative and post-operative test results. In developing countries, where gene testing is not frequently done due to resource and finance limitations, the root behind the same remains idiopathic. In our case, the cause for the same is yet to be elucidated and needs further discussion.
